# Key-Protease Inhibition Regimens Promote Tumor Targeting of Neurotensin Radioligands

**DOI:** 10.3390/pharmaceutics12060528

**Published:** 2020-06-09

**Authors:** Panagiotis Kanellopoulos, Aikaterini Kaloudi, Marion de Jong, Eric P. Krenning, Berthold A. Nock, Theodosia Maina

**Affiliations:** 1 Molecular Radiopharmacy, INRASTES, NCSR “Demokritos”, 15341 Athens, Greece; kanelospan@gmail.com (P.K.); katerinakaloudi@yahoo.gr (A.K.); nock_berthold.a@hotmail.com (B.A.N.); 2 Molecular Pharmacology, School of Medicine, University of Crete, 70013 Heraklion, Greece; 3 Department of Radiology & Nuclear Medicine Erasmus MC, 3015 CN Rotterdam, The Netherlands; m.hendriks-dejong@erasmusmc.nl; 4 Cyclotron Rotterdam BV, Erasmus MC, 3015 CE Rotterdam, The Netherlands; erickrenning@gmail.com

**Keywords:** neurotensin, neurotensin subtype 1 receptor, ^99m^Tc-radiotracer, tumor targeting, protease-inhibition, neprilysin-inhibitor, angiotensin-converting enzyme-inhibitor

## Abstract

Neurotensin subtype 1 receptors (NTS1R) represent attractive molecular targets for directing radiolabeled neurotensin (NT) analogs to tumor lesions for diagnostic and therapeutic purposes. This approach has been largely undermined by the rapid in vivo degradation of linear NT-based radioligands. Herein, we aim to increase the tumor targeting of three ^99m^Tc-labeled NT analogs by the in-situ inhibition of two key proteases involved in their catabolism. DT1 ([N_4_-Gly^7^]NT(7-13)), DT5 ([N_4_-*β*Ala^7^,Dab^9^]NT(7-13)), and DT6 ([N_4_-*β*Ala^7^,Dab^9^,Tle^12^]]NT(7-13)) were labeled with ^99m^Tc. Their profiles were investigated in NTS1R-positive colon adenocarcinoma WiDr cells and mice treated or not with the neprilysin (NEP)-inhibitor phosphoramidon (PA) and/or the angiotensin converting enzyme (ACE)-inhibitor lisinopril (Lis). Structural modifications led to the partial stabilization of ^99m^Tc-DT6 in peripheral mice blood (55.1 ± 3.9% intact), whereas ^99m^Tc-DT1 and ^99m^Tc-DT5 were totally degraded within 5 min. Coinjection of PA and/or Lis significantly stabilized all three analogs, leading to a remarkable enhancement of tumor uptake for ^99m^Tc-DT1 and ^99m^Tc-DT5, but was less effective in the case of poorly internalizing ^99m^Tc-DT6. In conclusion, NEP and/or ACE inhibition represents a powerful tool to improve tumor targeting and the overall pharmacokinetics of NT-based radioligands, and warrants further validation in the field of NTS1R-targeted tumor imaging and therapy.

## 1. Introduction

Recent advances in nuclear medicine include personalized treatment of cancer patients, whereby molecular probes are administered to deliver diagnostic or therapeutic radionuclides to cancer cells with high specificity. For example, peptide analogs may be used as carriers of gamma (^99m^Tc, ^111^In) or positron emitters (^68^Ga, ^64^Cu) for diagnostic single photon emission computed tomography (SPECT) or positron emission tomography (PET) imaging to identify patients eligible for radionuclide therapy. Therapy per se is then operated by the peptide analog carrying the respective therapeutic radionuclide (a beta, ^177^Lu, ^90^Y, Auger electron, ^111^In, or an alpha emitter, ^225^Ac) to upload cytotoxic radiation doses selectively to tumor lesions [1,2,3,4,5]. The rationale of this integrated “theranostic” approach relies on the overexpression of the target biomolecule in cancer cells while healthy surrounding tissues remain devoid of target expression [6].

Accordingly, the neurotensin subtype 1 receptor (NTS1R) [7,8] has been regarded as an attractive molecular target for such purposes owing to its high density expression in many human cancers [9,10]. Thus, high NTS1R expression has been documented in pancreatic ductal adenocarcinoma [11,12,13], Ewing’s sarcoma [14], colon carcinoma [15], prostate [16] and breast cancer [17], as well as other cancer types [18]. This finding offers the opportunity to develop theranostic NTS1R-directed radioligands based on the native tridecapeptide neurotensin (NT) for oncological applications. In recent years, many such analogs have been developed, whereby the C-terminal hexapeptide NT(8-13) representing the shortest fragment retaining full binding affinity for the NTS1R [19], served as motif. Several metal-chelators were coupled to its N-terminus, either directly or via different linkers, to accommodate a variety of clinical relevant radiometals [20,21,22,23,24,25,26,27,28,29].

Preclinical evaluation of resulting radiotracers revealed their propensity to proteolytic degradation, impairing their tumor targeting in animal models. Further NTS1R-directed radiotracers were designed by structural changes in the NT(8-13) motif, such as key-amino acid substitutions and/or manipulation of peptide bonds, like reduction, methylation or introduction of triazoles, aiming to metabolic stability improvements [30,31,32,33,34]. It should be noted however that radioligand stability was assessed mainly by the in vitro incubation of analogs in mice or human serum and not in vivo. Furthermore, these structural interventions often led to suboptimal pharmacokinetics, especially with regards to unfavorable increases of kidney uptake. Eventually, three NTS1R-radioligands were selected for clinical validation, but the overall results in patients turned out to be disappointing presumably as a result of fast in vivo catabolism [35,36,37].

Three cleavage sites have been identified in NT and NT(8-13), including the Arg^8^-Arg^9^ bond, the Pro^10^-Tyr^11^ bond and the Tyr^11^-Ile^12^ bond. A number of proteases have been shown to participate in the fast in-vivo catabolism of these peptides and their analogs. Thus, the angiotensin converting enzyme (ACE) is involved in the rapid cleavage of the Tyr^11^-Ile^12^ bond, whereas neprilysin (NEP) cleaves both the Pro^10^-Tyr^11^ and the Tyr^11^-Ile^12^ bonds [38,39,40,41,42]. It should be noted that NEP is an ectoenzyme present in high densities in many body tissues including vasculature epithelial cells [43,44]. Given that in plasma and serum NEP is practically absent, its impact on NTS1R-radioligand stability cannot be reliably assessed by in vitro incubation studies in the abovementioned biological fluids but should rather be conducted in vivo [33].

We have recently proposed a new strategy to improve the metabolic stability and hence the tumor targeting of radioligands from several peptide families via in situ inhibition of key-proteases involved in their catabolism. Protease-inhibition may be achieved by the co-administration of selected inhibitors together with the biodegradable radiopeptide and has led to marked enhancement of tumor uptake in animal models and most importantly also in human [45,46,47,48,49].

**Figure 1:**
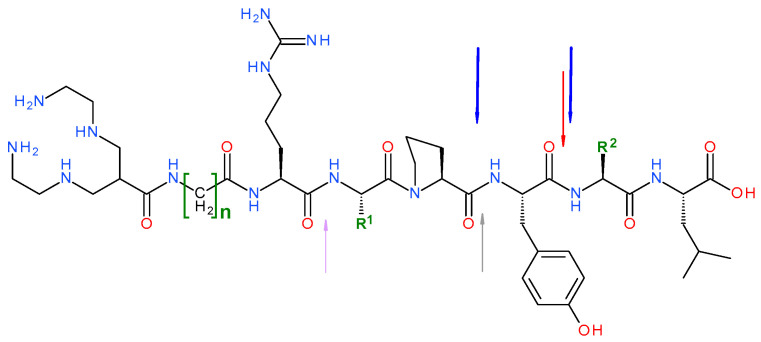
Structure of DT1 (n = 1, Gly; R^1^ = (CH_2_)_3_-NH-C=NH-NH_2_, Arg; R^2^ = CH(CH_3_)CH_2_CH_3_, Ile); DT5 (n = 2, *β*Ala; R^1^ = (CH_2_)_2_-NH_2_, Dab; R^2^ = CH(CH_3_)CH_2_CH_3_, Ile) and DT6 (n = 2, *β*Ala; R^1^ = (CH_2_)_2_-NH_2_, Dab; R^2^ = C(CH_3_)_3_, Tle); arrows indicate peptidase cleavage sites, violet: EC 3.4.24.15, gray: EC 3.4.24.16, blue: NEP and red: ACE.

We have decided to explore the efficacy of this promising strategy for the first time in the field of NTS1R-radiopeptides. For this purpose, herein, we compare the biological profile of three acyclic tetraamine-coupled NT(7-13) analogs suitable for labeling with the SPECT radionuclide ^99m^Tc (Figure 1), namely DT1 ([N_4_-Gly^7^]NT(7-13)), DT5 ([N_4_-*β*Ala^7^,Dab^9^]NT(7-13)), and DT6 ([N_4_-*β*Ala^7^,Dab^9^,Tle^12^]]NT(7-13)). Although the synthesis and biological data for these analogs in NTS1R-expressing cells and animal models were previously reported [23,24], in the present study, we mainly focus on the impact of in situ NEP and/or ACE inhibition on the in vivo stability and pharmacokinetic profile of these structurally related analogs. Protease inhibition has been achieved by treatment of mice with the NEP-inhibitor phosphoramidon (PA) [50] and/or the ACE inhibitor lisinopril (Lis) [51]. Findings from this study are discussed in relation to structural modifications of the NT(8-13) motif.

## 2. Materials and Methods

### 2.1. Peptide Analogs – Protease Inhibitors

The DT1 (N_4_-Gly-Arg-Arg-Pro-Tyr-Ile-Leu-OH, N_4_ = 6-(carboxy)-1,4,8,11-tetraazaundecane), DT5 (N_4_-*β*Ala-Arg-Dab-Pro-Tyr-Ile-Leu-OH) and DT6 (N_4_-*β*Ala-Arg-Dab-Pro-Tyr-Tle-Leu-OH) peptide conjugates (Figure 1) synthesized on the solid support as previously reported [23,24] were provided by PiChem (Graz, Austria). Neurotensin (NT = Pyr-Leu-Tyr-Glu-Asn-Lys-Pro-Arg-Arg-Pro-Tyr-Ile-Leu-OH) was purchased from Bachem (Bubendorf, Switzerland). The NEP-inhibitor PA (phosphoramidon disodium dehydrate, *N*-(α-rhamnopyranosyloxy-hydroxyphosphinyl)-l-leucyl-l-tryptophan × 2Na × 2H_2_O) was obtained from PeptaNova GmbH (Sandhausen, Germany) and the ACE-inhibitor Lis (lisinopril dehydrate, ((S)1–1-[N2-(1-carboxy-3-phenylpropyl)-lysyl-proline dehydrate, MK 521) from Sigma–Aldrich (St. Louis, MO, USA).

#### Preparation and Quality Control of ^99m^Tc-DT1, ^99m^Tc-DT5 and ^99m^Tc-DT6

Each of lyophilized DT1, DT5 or DT6 was dissolved in HPLC-grade H_2_O (2 mg/mL) and 50 μL aliquots thereof were stored in Eppendorf Protein LoBind tubes at −20 °C. Labeling with ^99m^Tc was conducted in an Eppendorf vial, wherein the following solutions were consecutively added: i) 0.5 M phosphate buffer pH 11.5 (50 μL), ii) 0.1 M sodium citrate (5 μL), iii) [^99m^Tc]NaTcO_4_ generator eluate (415 mL, 300–500 MBq; Ultra-Technekow™ V4 Generator, Curium Pharma), iv) peptide conjugate stock solution (15 μL, 15 nmol) and v) freshly made SnCl_2_ solution in EtOH (30 μg, 15 μL). After reaction for 30 min at ambient temperature, the pH was brought to ~7 by adding 1 M HCl (10 μL).

Reversed-phase HPLC was performed on a Waters Chromatograph based on a 600E multisolvent delivery system coupled to a Waters 2998 photodiode array detector and a Gabi gamma-detector (Raytest, RSM Analytische Instrumente GmbH, Straubenhardt, Germany). Data processing and chromatography were controlled by the Empower Software (Waters, Vienna, Austria). For quality control aliquots of the radiolabeling solution a Symmetry Shield RP18 cartridge column (5 μm, 3.9 × 20 mm, Waters) was used for analyses. Solutes were eluted with 0.1% TFA/MeCN applying a linear gradient starting from 0% MeCN and a 2% increase per min at 1 mL/min flow rate (system 1). TLC analysis was additionally performed on Whatman 3 mm chromatography paper strips (GE Healthcare, Chicago, IL, USA), developed up to 10 cm from the origin with 5 M ammonium acetate/MeOH 1/1 (*v*/*v*) for ^99m^TcO_2_ × *n*H_2_O, or acetone for ^99m^TcO_4_^−^ detection [23,24]. The radiochemical labeling yield exceeded 98% and the radiochemical purity was >99%. Samples of ^99m^Tc-DT1, ^99m^Tc-DT5 and ^99m^Tc-DT6 were tested before and after the end of biological experiments.

All manipulations with beta and gamma emitting radionuclides and their solutions were performed by trained and authorized personnel behind suitable shielding in licensed laboratories in compliance to European radiation-safety guidelines and supervised by the Greek Atomic Energy Commission (license # A/435/17092/2019).

### 2.2. In Vitro Assays

#### 2.2.1. Cell Lines and Culture

Human colorectal adenocarcinoma WiDr cells (LGC Promochem, Teddington, UK) expressing the human NTS1R [52] were cultured in McCoy’s GLUTAMAX-I (Gibco BRL, Life Technologies, Grand Island, NY, USA) supplemented by 10% (*v*/*v*) fetal bovine serum (FBS), 100 U/mL penicillin and 100 μg/mL streptomycin (all from Biochrom KG Seromed, Berlin, Germany). Cells were kept in a controlled humidified atmosphere containing 5% CO_2_ at 37 °C. Passages were performed every 4–5 days using a trypsin/EDTA (0.05%/0.02% *w*/*v*) solution.

#### 2.2.2. Internalization Assay in WiDr Cells

The overall cell association–internalization of ^99m^Tc-DT1, ^99m^Tc-DT5 and ^99m^Tc-DT6 was assessed in confluent WiDr cells. Briefly, the cells were seeded in six-well plates (~1 × 10^6^ cells per well) 24 h before the experiment. Each of ^99m^Tc-DT1, ^99m^Tc-DT5 and ^99m^Tc-DT6 (approximately 150,000 cpm, 250 fmol total peptide in 150 μL of 0.5% BSA/PBS) was added alone (total) or in the presence of 1 μM NT (non-specific). Cells were incubated at 37 °C for 1 h and incubation was interrupted by placing the plates on ice, removing the supernatants and rapid rinsing with ice-cold 0.5% BSA/PBS. Cells were then treated 2 × 5 min with acid wash buffer (2 × 0.6 mL, 50 mM glycine buffer, pH 2.8, 0.1 M NaCl) at room temperature and supernatants were collected (membrane-bound fraction). After rinsing with 1 mL chilled 0.5% BSA/PBS, cells were lyzed by treatment with 1 N NaOH (2 × 0.6 mL) and lysates were collected (internalized fraction). Sample radioactivity was measured in the γ-counter (an automated well-type gamma counter with a NaI(Tl) 3′′ crystal, Canberra Packard Auto-Gamma 5000 series instrument) and total cell-associated (internalized + membrane bound) radioactivity was determined vs. total added activity. Results represent the average values ± sd of 3 experiments in triplicate.

### 2.3. Animal Studies

All animal studies were performed in compliance to European guidelines in supervised and licensed facilities (EL 25 BIO 021), whereas the study protocols were approved by the Department of Agriculture and Veterinary Service of the Prefecture of Athens (protocol numbers # 1609 for the stability studies and # 1610 for biodistribution and imaging studies).

#### 2.3.1. In Vivo Stability Tests

For stability experiments, healthy male Swiss albino mice (30 ± 5 g, NCSR “Demokritos” Animal House Facility) were used. Each radioligand, ^99m^Tc-DT1, ^99m^Tc-DT5, or ^99m^Tc-DT6, was injected as a 100 μL bolus (50–60 MBq, 3 nmol total peptide in vehicle: saline/EtOH 9/1 *v*/*v*) in the tail vein together with vehicle (100 μL; control) or with i) a phosphoramidon (PA)-solution (100 μL injection solution containing 300 μg PA), ii) a Lis solution (100 μL injection solution containing 200 μg Lis), or iii) a solution of both inhibitors (100 μL injection solution containing 300 μg PA plus 200 μg Lis). Animals were euthanized and blood (0.5–1 mL) was directly withdrawn from the heart and transferred in a pre-chilled EDTA-containing Eppendorf tube on ice. Blood samples were centrifuged for 10 min at 2000× *g*/4 °C and plasma was collected. After addition of an equal volume of ice-cold MeCN the mixture was centrifuged for 10 min at 15,000× *g* and 4 °C. The supernatant was concentrated under a N_2_-flux at 40 °C to 0.05–0.1 mL, diluted with saline (0.4 mL), filtered through a 0.22 μm Millex GV filter (Millipore, Milford, MA, USA) and analyzed by RP-HPLC. The Symmetry Shield RP18 (5 μm, 3.9 mm × 20 mm) column was eluted at a flow rate of 1.0 mL/min applying a linear gradient starting with 0% B at 0 min and reaching to 30% B in 30 min; A = 0.1 % TFA in water and B = MeCN (system 2). The *t*_R_ of the intact radiopeptide was determined by coinjection with the respective ^99m^Tc-DT1, ^99m^Tc-DT5, or ^99m^Tc-DT6 reference in the HPLC.

#### 2.3.2. Induction of WiDr Xenografts in Mice

A suspension containing freshly harvested human WiDr cells (≈150 μL of a ≈1.8 × 10^7^ cells) was subcutaneously injected in the flanks of male severe combined immune deficiency (SCID) mice (20 ± 3 g, six weeks of age at the day of arrival, NCSR “Demokritos” Animal House Facility). The animals were kept under aseptic conditions and 3 weeks later developed well-palpable tumors (100–300 mg) at the inoculation sites.

#### 2.3.3. Biodistribution of ^99m^Tc-Radiotracers in WiDr Xenograft-Bearing SCID Mice

For the biodistribution study, animals in groups of 4 received via the tail vein a 100 μL bolus of ^99m^Tc-DT1, ^99m^Tc-DT5 or ^99m^Tc-DT6 (180–230 kBq, corresponding to 10 pmol total peptide) co-injected either with injection solution (100 μL; control) or i) a PA-solution (^99m^Tc-DT1 and ^99m^Tc-DT6), or ii) a Lis-solution (^99m^Tc-DT1 and ^99m^Tc-DT6), iii) a PA plus Lis-solution (^99m^Tc-DT1, ^99m^Tc-DT5 and ^99m^Tc-DT6), or iv) an excess NT (100 μL injection solution containing 100 μg NT for in vivo NTS1R-blockade along with PA+Lis); ^99m^Tc-DT1, or ^99m^Tc-DT6). Animals were euthanized at 4 h post-injection (pi) and dissected. Samples of blood, tumors, and organs of interest were collected, weighed, and measured for radioactivity in the gamma counter. Intestines and stomach were not emptied of their contents. Data was calculated as percent injected dose per gram tissue (%ID/g) with the aid of standard solutions and represent mean values ± sd, *n* = 4.

#### 2.3.4. Statistical Analysis

For statistical analysis of biological results, the two-way ANOVA with multiple comparisons was used applying Tukey’s post hoc analysis (GraphPad Prism Software, San Diego, CA, USA). *P* values of <0.05 were considered to be statistically significant. For cell-association experiments, the one-way ANOVA with Tukey’s post hoc analysis was used instead.

#### 2.3.5. SPECT/CT Imaging of ^99m^Tc-DT1 in WiDr Xenograft-Bearing SCID Mice

For SPECT/CT imaging, three mice bearing WiDr xenografts were injected in the tail vein with a bolus containing ^99m^Tc-DT1 (100 μL, ≈50 MBq, 1.5 nmol total peptide, in vehicle) together with vehicle (100 μL; controls) or with a PA+Lis solution or with a PA+Lis solution in gelofusin (100 μL; Gelo) and were euthanized at 3 h pi. Tomographic SPECT/CT imaging was performed with the y-CUBE/x-CUBE systems (Molecubes, Belgium). The SPECT system is based on monolithic NaI detectors attached to SiPMs, with a 0.6 mm intrinsic resolution. The CT system is based on a structured CMOS detector of CsI with pixels of 75 μm and operates between 35 and 80 kVp, 10–500 μA tube current, with a 33 μm fixed focal spot size. SPECT scans were acquired over 3 h pi, with a 30 min duration protocol based on the injected activity and each SPECT scan was succeeded by a CT scan, following a general purpose protocol under 50 kVp, for co-registration purposes. SPECT images were reconstructed by the MLEM reconstruction method with a 250 μm voxel size and 500 iterations. CT images were reconstructed by using the ISRA reconstruction method with a 100 μm voxel size. Images were exported and post-processed on VivoQuant software, version 4.0 (Invicro, Boston, MA, USA). A smoothing median filter (0.6 mm, spherical window) was applied to the images and bladder was removed for consistency purposes.

## 3. Results

### 3.1. Peptides and Radioligands

Labeling of DT1, DT5 and DT6 with ^99m^Tc typically proceeded by brief peptide-conjugate incubation with ^99m^TcO_4_^−^ generator eluate, SnCl_2_ as reducing agent and citrate anions as transfer ligand in alkaline pH at ambient temperature and molecular activities of 20–40 MBq ^99m^Tc/nmol peptide. Quality control of the radiolabeled products combined HPLC and TLC analysis. The total radiochemical impurities, comprising ^99m^TcO_4_^−^, [^99m^Tc]citrate and ^99m^TcO_2_ × *n*H_2_O, did not exceed 2%, while a single radiopeptide species was detected by RP-HPLC. In view of >98% labeling yields and >99% radiochemical purity of the resultant ^99m^Tc-DT1, ^99m^Tc-DT5 or ^99m^Tc-DT6, the radioligands were used without further purification in all subsequent experiments. 

### 3.2. In Vitro Assays

#### Comparative Internalization of ^99m^Tc-DT1, ^99m^Tc-DT5 or ^99m^Tc-DT6 in WiDr Cells

**Figure 2:**
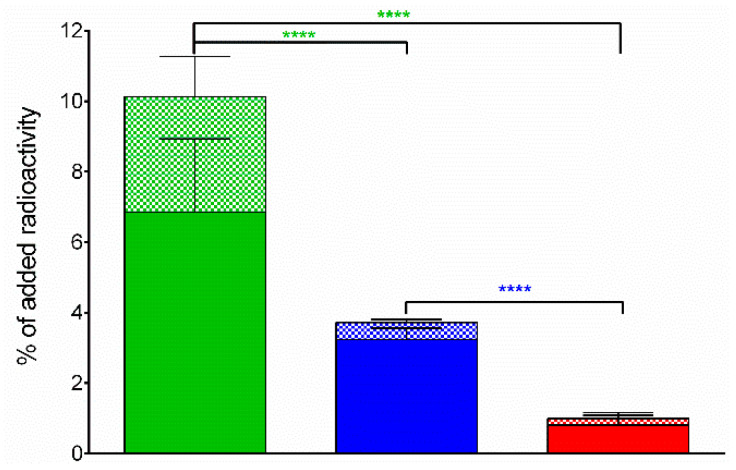
NTS1R-specific cell uptake of ^99m^Tc-DT1 (green bars), ^99m^Tc-D5 (blue bars) and ^99m^Tc-DT6 (red bars) in WiDr cells during 1 h incubation at 37 °C (solid bars: internalized fraction; checkered bars: membrane bound fraction). Results represent average values ± sd (*n* = 3, in triplicate); non-specific values were obtained in the presence of 1 μM NT and were subtracted from totals to provide specific values; the study was conducted with WiDr cells as confluent monolayers. Statistical analysis is shown for whole-cell association with **** representing *P* < 0.0001.

During 1 h incubation at 37°C, ^99m^Tc-DT1, ^99m^Tc-DT5 or ^99m^Tc-DT6 were taken up by WiDr cells via an NTS1R-mediated process, as shown by the lack of uptake observed in the presence of excess NT (results not shown). In all cases, the bulk of cell-bound radioactivity was found in the internalized fraction, as consistent with a radioagonist profile. The radioligand rank of cell uptake/internalization was ^99m^Tc-DT1 (10.1 ± 2.3%/6.9 ± 2.1%) >> ^99m^Tc-DT5 (3.7 ± 0.3%/3.2 ± 0.3%) > ^99m^Tc-DT6 (1.0 ± 0.4%/0.8 ± 0.4%), revealing the negative effect of structurally modifying ^99m^Tc-DT1 on cell uptake (Figure 2).

### 3.3. In Vivo Comparison of ^99m^Tc-DT1, ^99m^Tc-DT5 or ^99m^Tc-DT6

#### 3.3.1. Stability of ^99m^Tc-DT1, ^99m^Tc-DT5 and ^99m^Tc-DT6 in Mice

**Figure 3:**
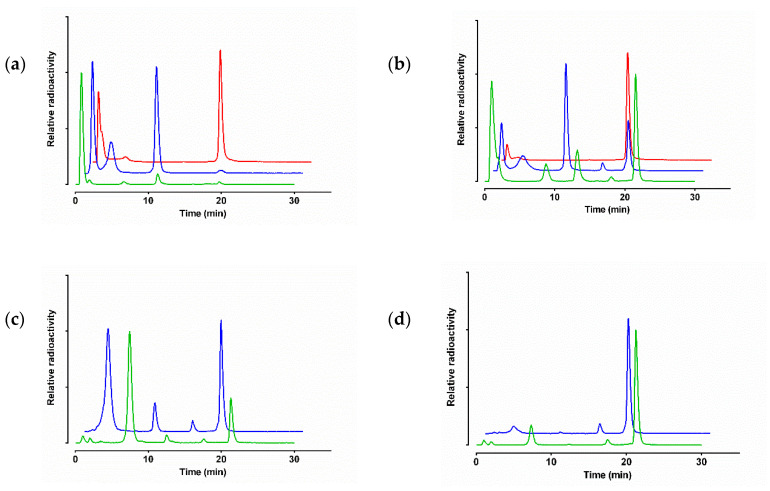
Representative radiochromatograms of HPLC analysis of mouse blood samples collected 5 min pi of ^99m^Tc-DT1 (green line), ^99m^Tc-DT5 (blue line) or ^99m^Tc-DT6 (red line) administered (**a**) without, or (**b**) with PA-, or (**c**) with Lis-, or (**d**) with PA+Lis-coinjection (HPLC system 2); percentages of intact radioligand are summarized in Table 1.

The radiotracers exhibited distinct resistance to degrading proteases after injection in mice. As revealed by HPLC analysis of blood samples collected at 5 min pi ^99m^Tc-DT1 and ^99m^Tc-DT5 were found equally fast degraded (1.8 ± 0.8% and 1.3 ± 0.2% intact; *P* > 0.05) whereas the Tle^12^-analog ^99m^Tc-DT6 displayed significantly higher stability (55.1 ± 3.9% intact; *P* < 0.0001) (Table 1; representative radiochromatograms are shown in Figure 3).

It should be noted that coinjection of the NEP-inhibitor PA enhanced the in vivo stability of all three radioligands, implicating NEP in their degradation. PA was particularly effective in stabilizing ^99m^Tc-DT6 in mice blood, showing NEP as the major degrading protease of the radiotracer.

On the other hand, coinjection of the ACE-inhibitor Lis enhanced the in vivo stability of ^99m^Tc-DT1 and ^99m^Tc-DT5, revealing the role of this second protease in their in vivo degradation. It is interesting to note that treatment of mice with both protease-inhibitors resulted in a further rigorous enhancement of metabolic stability in peripheral mice blood (72.3 ± 3.2% and 79.0 ± 6.7% intact, respectively; *P* < 0.0001). 

#### 3.3.2. Biodistribution of ^99m^Tc-DT1, ^99m^Tc-DT5 and ^99m^Tc-DT6 in WiDr Xenograft-Bearing Mice 

The biodistribution of ^99m^Tc-DT1, ^99m^Tc-DT5 and ^99m^Tc-DT6 was studied at 4 h pi in SCID mice bearing subcutaneous WiDr tumors expressing the human NTS1R. Cumulative biodistribution results for ^99m^Tc-DT1, ^99m^Tc-DT5 and ^99m^Tc-DT6 are summarized in Table 2, Table 3 and Table 4, respectively, and are expressed as mean %ID/g ± sd, *n* = 4. All three radiotracers washed rapidly from the blood and the body of mice predominantly via the kidneys and the urinary system in the control animal groups. However, the Dab^9^-modified analogs ^99m^Tc-DT5 (8.63 ± 1.8%ID/g; *P* < 0.001) and ^99m^Tc-DT6 (6.09 ± 1.15%ID/g; *P* < 0.01) showed significantly higher renal retention compared to ^99m^Tc-DT1 (1.80 ± 0.14%ID/g). Uptake in the intestines was comparable across radioligands (*P* > 0.05) and could be partially blocked by excess NT, given that intestines were not emptied of their contents and NTS1R are expressed in the intestinal walls [53]. Likewise, uptake in the implanted tumors was comparable across analogs (*P* > 0.05).

Treatment of mice with PA and Lis resulted in remarkable enhancement of tumor uptake of ^99m^Tc-DT1 (from 1.20 ± 0.21%ID/g to 9.60 ± 3.62%ID/g; *P* < 0.0001) and ^99m^Tc-DT5 (from 0.88 ± 0.08%ID/g to 12.29 ± 2.73%ID/g; *P* < 0.0001) and only to a lesser, but not in statistically significant extent in the case of ^99m^Tc-DT6 (from 1.68 ± 0.28%ID/g to 3.50 ± 0.34%ID/g; *P* > 0.05). This enhancement is compromised for both ^99m^Tc-DT5 and ^99m^Tc-DT6 by a further unfavorable increase of the already elevated renal accumulation. It is interesting to observe that for ^99m^Tc-DT6 the PA alone suffices to achieve the highest tumor uptake (PA and PA+Lis tumor values *P* > 0.05), while both inhibitors are required for maximum in vivo stabilization and highest tumor values for ^99m^Tc-DT1 and ^99m^Tc-DT5.

**Figure 4:**
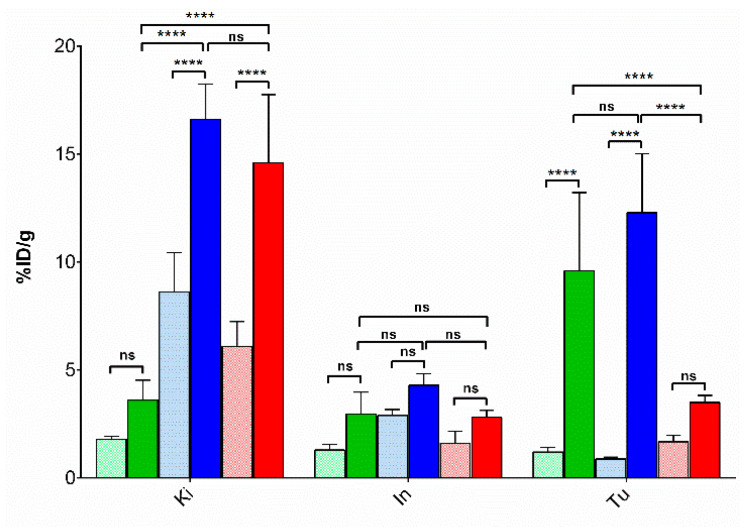
Comparative uptake for ^99m^Tc-DT1 (green bars), ^99m^Tc-DT5 (blue bars) or ^99m^Tc-DT6 (red bars) in kidneys, intestines and WiDr-xenografts at 4 h pi of radioligand administration without (paler bars), or with PA+Lis-coinjection (darker bars) in male SCID mice; results are given as mean %ID/g values ± sd, *n* = 4. Statistical analysis results are also incorporated in the diagram, with **** representing *P* < 0.0001 and ns (non-significant) *P* > 0.05.

Comparative biodistribution results for ^99m^Tc-DT1, ^99m^Tc-DT5, and ^99m^Tc-DT6 in mice kidneys, intestines and WiDr tumor 4 h after radioligand administration without or with coinjection of PA+Lis are selectively depicted in Figure 4. The favorable increase in the tumor uptake of ^99m^Tc-DT1 is not compromised by a high increase of renal accumulation during PA+Lis treatment, as seen for the Dab^9^-modified ^99m^Tc-DT5. On the other hand, ^99m^Tc-DT6, despite its high in vivo stabilization by this regimen, fails to reach similar levels of tumor uptake, most probably due to its poor in vitro internalization capability. At the same time, renal accumulation has unfavorably increased.

#### 3.3.3. SPECT/CT of ^99m^Tc-DT1 in WiDr Xenograft-Bearing Mice

**Figure 5:**
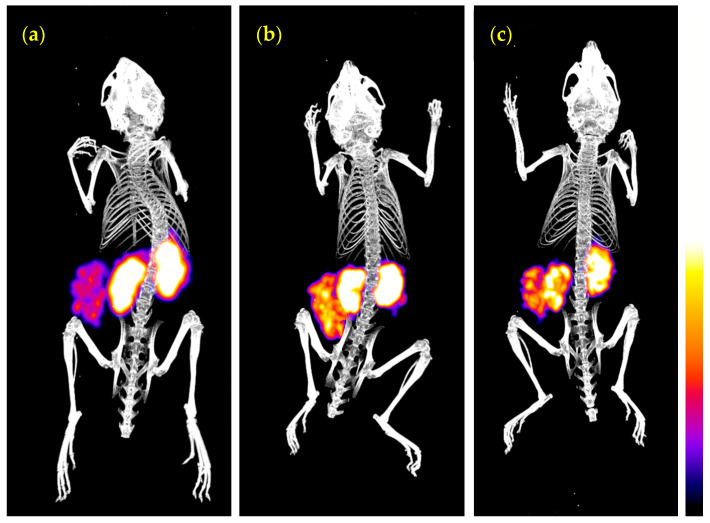
Static whole body SPECT/CT images of three SCID mice bearing WiDr tumors 3 h after injection of ^99m^Tc-DT1 (**a**) alone, or (**b**) with coinjection of PA+Lis, or (**c**) with coinjection PA+Lis in gelofusine. Intense uptake is seen on kidneys and tumor. In (**a**) high uptake is observed in the kidneys and in (**b**) a notable increase in the tumor uptake has resulted after treatment of mice with PA+Lis. Kidneys and tumor present a very similar uptake in (**c**), with the renal uptake being markedly reduced compared with (**b**) due to gelofusine coinjection. The color bar indicates the difference in accumulated activity (purple being the lowest and white the highest level of accumulation).

Mice SPECT/CT images obtained 3 h after injection of ^99m^Tc-DT1 are presented on Figure 5. Significant accumulation was achieved in the NTS1R-expressing WiDr xenografts and the kidneys. Clear differences in renal and tumor accumulation could be observed between control (Figure 5a), PA+Lis treated (Figure 5b) and animals coinjected with PA+Lis in the kidney protecting agent, gelofusine (Figure 5c).

## 4. Discussion

Radiolabeled analogs of neurotensin and especially its C-terminal hexapeptide fragment, NT(8-13), have been proposed for diagnostic imaging and radionuclide therapy of human tumors with high NTS1R expression. In search of metabolically robust analogs, a variety of structural modifications have been introduced on the linear peptide NT(8-13) motif and their impact on metabolic stability, tumor targeting, and overall pharmacokinetics have been assessed during structure–activity relationships studies [9,10,18,19,30,34]. The degradation of NT, NT(8-13), and their analogs has been studied in the past, revealing three major cleavage sites on the peptide backbone: i) Arg^8^-Arg^9^, ii) Pro^10^-Tyr^11^ and iii) Tyr^11^-Ile^12^. Amongst the peptidases participating in the rapid in vivo catabolism of these analogs, NEP (cleaving the Pro^10^-Tyr^11^ and the Tyr^11^-Ile^12^ bonds) and ACE (cleaving the Tyr^11^-Ile^12^ bond) play a major role. Two additional metallopeptidases, EC 3.4.24.15 (thiolsensitive metallo oligopeptidase, thimet-oligopeptidase, TOP) hydrolyzing the Arg^8^-Arg^9^ bond and EC 3.4.24.16 (neurolysin) hydrolyzing the Pro^10^-Tyr^11^ bond (Figure 1), are also implicated in the degradation of NT, NT(8-13) and their analogs [30,31,33,34,38,40,41,42]. However, the latter two enzymes are predominantly located within the cells and hence are less expected to have any considerable impact on the stability of circulating NT-derived radiopeptides after iv administration and on their way to tumor sites [54,55].

Thus far, most efforts toward metabolically stable NT-analogs and their radioligands have been focused on the two Arg^8^-Arg^9^ and Tyr^11^-Ile^12^ bonds. Stabilization of the Arg^8^-Arg^9^ bond has been attempted by a replacement of either Arg by DArg, Lys, or Dab as well as by a reduction or methylation of the bond. Further stabilization of the Tyr^11^-Ile^12^ site could be achieved by substitution of Ile^12^ by Tle^12^ [30,31,33,34,41]. The effects of the above modifications on the pharmacokinetic profile of resulting radioligands have not been fully understood. This may be attributed to the methods commonly applied for metabolic stability determination, comprising almost exclusively in vitro radioligand incubation in tissue homogenates and/or serum/plasma [30,33,34]. In the first case, homogenization will disrupt cell membranes and otherwise intracellularly compartmentalized enzymes will be released. As a result, radioligands will be exposed to enzymes which they would not actually encounter after injection in the living organism. On the other hand, during in vitro incubation of radiopeptides in serum or plasma, the action of ectoenzymes anchored on epithelial cells of the vasculature and other tissues of the body, e.g., NEP [42,43,44], is totally overseen [33,45]. 

We have previously developed a series of NT(8-13)-based analogs carrying an open chain tetraamine chelator for stable binding of ^99m^Tc [23,24]. During incubation of resulting ^99m^Tc-radioligands in mice plasma, we have also observed increasing stabilization effects by Arg^9^/Dab^9^ and Ile^12^/Tle^12^ replacements. However, responses on overall pharmacokinetics in tumor-bearing mice were less straightforward. For example, renal accumulation unfavorably increased without any remarkable gain in tumor targeting for ^99m^Tc-DT6, as would be expected by the high metabolic in vitro stability determined for this doubly substituted, Arg^9^/Dab^9^ and Ile^12^/Tle^12^, analog. Similar sub-optimal tumor-targeting results were subsequently obtained in a pilot study with ^99m^Tc-DT6 in a small number of cancer patients [36]. 

Aiming to better understand the above intriguing findings, we herein compare the biological profiles of such previously reported NT-radiopeptides under a new light. We achieve this mainly by taking into account results from recent studies relating in vivo metabolic fate and tumor targeting capabilities of a series of biodegradable radiopeptides [45,46,47,48,49]. These studies have revealed the central, but hitherto unrecognized, role of NEP in the rapid catabolism of many radiopeptides from different peptide families, including somatostatin, bombesin and gastrin, in peripheral blood, compromising their sufficient delivery to tumor sites. Most interestingly, by administration of suitable NEP-inhibitors it was possible to induce stabilization of these radiopeptides in circulation, thereby enhancing tumor localization with clear benefits envisaged for diagnosis and therapy. In the present study, we report for the first time, outcomes after applying this methodology in the field of NT-radiopeptides. For this purpose, we selected ^99m^Tc-DT1 (^99m^Tc-[N_4_-Gly^7^]NT(7-13)), ^99m^Tc-DT5 (^99m^Tc-[N_4_-*β*Ala^7^,Dab^9^]NT(7-13)) and ^99m^Tc-DT6 (^99m^Tc-[N_4_-*β*Ala^7^,Dab^9^,Tle^12^]]NT(7-13)) as examples (Figure 1).

Surprisingly, we observed gradual loss of cell uptake/internalization from ^99m^Tc-DT1 to ^99m^Tc-DT5 (single Arg^9^/Dab^9^-replacement) to ^99m^Tc-DT6 (double Arg^9^/Dab^9^ and Ile^12^/Tle^12^ replacement) in NTS1R-expressing WiDr cells, revealing a negative effect of above modifications on the interaction of resulting radioligands with the NTS1R at the cellular level. This result has been previously missed and was unexpected considering the comparable affinities of these analogs for the NTS1R [23,24]. 

On the other hand, only the double-modified ^99m^Tc-DT6 showed a significantly higher metabolic stability in peripheral mice blood at 5 min pi (55.1 ± 3.9% intact) compared with either ^99m^Tc-DT1 or ^99m^Tc-DT5 that were degraded within this period (1.8 ± 0.8% and 1.3 ± 0.2% intact, respectively; *P* < 0.0001). This finding demonstrates that single Arg^9^/Dab^9^ replacement had no impact on the stability of the circulating radioligand. It further shows that both ^99m^Tc-DT5 and ^99m^Tc-DT6 are more rapidly degraded in vivo compared to their in vitro degradation in mouse plasma [23]. To investigate the involvement of NEP or ACE in the rapid in vivo catabolism of all three radioligands, we have treated mice with the NEP-inhibitor PA, the ACE-inhibitor Lis, or a combination of the two, and analyzed the blood of mice collected 5 min afterwards with HPLC. As summarized in Table 1, PA co-injection led to maximum stabilization of double-modified ^99m^Tc-DT6 (89.3 ± 6.7% intact; *P* < 0.0001). Considering the high in vitro stability of ^99m^Tc-DT6 in mice plasma, whereby NEP is absent and ACE is present, this result implies that NEP plays the major role in the in vivo degradation of the Ile^12^/Tle^12^-modified radioligand. In contrast, ^99m^Tc-DT1 or ^99m^Tc-DT5 were only partially stabilized by either PA or Lis and only their combination led to maximum stabilization in peripheral mouse blood (72.3 ± 3.2% intact and 79.0 ± 1.7% intact, respectively; *P* < 0.0001). This result suggests a combined role for ACE and NEP in the in vivo degradation of NT-radioligands non-substituted at Ile^12^ [33,38,39,40].

It is interesting to observe how the abovementioned structural modifications, affecting both internalization/cell-uptake of the three radioligands and their in vivo stability, translate in NTS1R-specific tumor localization and overall pharmacokinetics in WiDr tumor-bearing mice. As shown in Table 2, Table 3 and Table 4 and Figure 4, there’s no significant difference in the localization of ^99m^Tc-DT1, ^99m^Tc-DT5 or ^99m^Tc-DT6 in the implanted tumors (*P* > 0.05). Thus, effects induced by structural changes compensated each other, eventually resulting in similar outcomes with regards to tumor uptake. In contrast, renal uptake is found significantly increased in both Arg^9^/Dab^9^-modified ^99m^Tc-DT5 (8.63 ± 1.80%ID/g; *P* < 0.0001) and ^99m^Tc-DT6 (6.09 ± 1.15%ID/g; *P* < 0.01) compared with ^99m^Tc-DT1 (1.80 ± 0.14%ID/g), revealing the unfavorable impact of the pendant -NH_2_ of Dab^9^ on kidney accumulation. Similar observations have been previously reported for other Lys^8^- or Lys^9^-modified NT-radioligands likewise carrying pendant -NH_2_ groups [23,24,34].

Of particular interest is the impact of in situ NEP- and ACE-inhibition on the biodistribution of these radioligands. After combined treatment of mice with PA and Lis, tumor localization significantly increased for both ^99m^Tc-DT1 (9.60 ± 3.62%ID/g; *P* < 0.0001) and ^99m^Tc-DT5 (12.29 ± 2.73%ID/g; *P* < 0.0001) compared with untreated controls, highlighting the significance of in vivo stability for the efficient delivery of intact radiopeptides to tumor sites, as previously documented [45,46,47,48]. Surprisingly, the increase observed in the tumor uptake of ^99m^Tc-DT6 was much lower (3.50 ± 0.34%ID/g; *P* > 0.05) indicating the pronounced negative impact of the poor internalization/cell uptake of this Ile^12^/Tle^12^-modified radiotracer on tumor uptake. On the other hand, renal accumulation was found to be increased for all analogs after PA+Lis treatment, but in the case of the two Arg^9^/Dab^9^-modified radioligands, renal values were significantly much higher compared with ^99m^Tc-DT1 (^99m^Tc-DT5: 16.62 ± 1.63%ID/g; *P* < 0.0001 and ^99m^Tc-DT6: 14.61 ± 3.16%ID/g; *P* < 0.0001 vs. ^99m^Tc-DT1: 3.61 ± 0.93%ID/g; *P* > 0.05). In view of the above, the most favorable pharmacokinetics amongst this series of NT-radiotracers are displayed by ^99m^Tc-DT1 during treatment of mice with the PA+Lis combination. In contrast, the structural interventions in ^99m^Tc-DT5 (high kidney accumulation) and ^99m^Tc-DT6 (poor cell association/internalization and consequent low tumor uptake), despite their stabilization effect, led to unfavorable overall pharmacokinetics.

## 5. Conclusions

The impact of in-situ NEP and ACE inhibition on tumor targeting and pharmacokinetics of NT-based radioligands has been assessed for the first time employing three structurally related analogs, namely ^99m^Tc-DT1 (^99m^Tc-[N_4_-Gly^7^]NT(7-13)), ^99m^Tc-DT5 (^99m^Tc-[N_4_-*β*Ala^7^,Dab^9^]NT(7-13)), and ^99m^Tc-DT6 (^99m^Tc-[N_4_-*β*Ala^7^,Dab^9^,Tle^12^]]NT(7-13)), as examples. Structural interventions led to sub-optimal outcomes, with Arg^9^/Dab^9^-replacement leading to unfavorable renal accumulation (^99m^Tc-DT5 and ^99m^Tc-DT6) and further Ile^12^/Tle^12^-substitution compromising cell uptake/internalization (^99m^Tc-DT6). Notably, ^99m^Tc-DT1, retaining high cell-binding/internalization capability and effectively stabilized in vivo by PA+Lis coinjection, achieved markedly enhanced tumor localization while preserving lower kidney values. These findings verify previous reports on the improved pharmacokinetic profile of other radiopeptides from different families in animal models and very recently in patients during key-protease inhibition. Hence, they highlight the need for further validation of this promising concept in the field of neurotensin radioligands in cancer theranostics. It should be noted that registered NEP and or ACE inhibitors have been used for years and can theoretically be applied in oncology. The first results of using such an over-the-counter NEP-inhibitor drug (racecadotril) in combination with biodegradable radiolabeled gastrin in medullary thyroid cancer patients have been recently reported [49] and strongly support the clinical applicability of this concept. 

## 6. Patents

Theodosia Maina, Berthold A. Nock, Marion de Jong Enhanced in vivo targeting of radiolabelled peptides with the means of enzyme inhibitors. EP2729183B1; 05.09.2018 Bulletin 2018/36.

## Figures and Tables

**Table 1:** Stabilities of ^99m^Tc-DT1, ^99m^Tc-DT5 and ^99m^Tc-DT6 in peripheral mouse blood 5 min pi.

	^99m^Tc-DT1	^99m^Tc-DT5	^99m^Tc-DT6
Control	1.8 ± 0.8 (*n* = 4)	1.3 ± 0.2 (*n* = 2)	55.1 ± 3.9 (*n* = 2)
PA	26.4 ± 2.2 (*n* = 2)	15.4 ± 5.1 (*n* = 2)	89.3 ± 6.7 (*n* = 4)
Lis	18.8 ± 2.5 (*n* = 3)	28.8 ± 5.2 (*n* = 2)	-
PA+Lis	72.3 ± 3.2 (*n* = 4)	79.0 ± 1.7 (*n* = 2)	-

Data represents the mean percentage of intact radioligand ± sd; *n* of experiments are shown in parentheses.

**Table 2:** Biodistribution data for ^99m^Tc-DT1, expressed as %ID/g mean ± sd, *n* = 4, in WiDr xenograft-bearing SCID mice at 4 h pi without or with coinjection of PA, Lis, or PA+Lis.

Tissue	^99m^Tc-DT1 – 4 h pi				
	Block ^1^	Controls	4 h PA ^2^	Lis ^3^	PA+Lis ^4^
Blood	0.07 ± 0.02	0.15 ± 0.01	0.12 ± 0.03	0.10 ± 0.01	0.13 ± 0.06
Liver	0.42 ± 0.02	0.42 ± 0.02	0.47 ± 0.05	0.39 ± 0.05	0.58 ± 0.12
Heart	0.05 ± 0.01	0.08 ± 0.00	0.10 ± 0.02	0.08 ± 0.02	0.10 ± 0.02
Kidneys	2.89 ± 0.56	1.80 ± 0.14	2.26 ± 0.30	2.24 ± 0.34	3.61 ± 0.93
Stomach	0.90 ± 0.25	1.89 ± 0.26	1.39 ± 0.12	1.18 ± 0.22	1.39 ± 0.73
Intestines	0.43 ± 0.14	1.30 ± 0.26	2.91 ± 0.30	1.72 ± 0.29	2.96 ± 1.02
Spleen	0.26±0.02	0.26 ± 0.06	0.56 ± 0.13	0.32 ± 0.07	1.01 ± 0.30
Muscle	0.02 ± 0.02	0.03 ± 0.01	0.05 ± 0.03	0.03 ± 0.01	0.07 ± 0.06
Lungs	0.23 ± 0.10	0.20 ± 0.03	0.42 ± 0.06	0.19 ± 0.02	0.67 ± 0.08
Pancreas	0.04 ± 0.01	0.08 ± 0.01	0.10 ± 0.02	0.06 ± 0.01	0.12 ± 0.02
Tumor	0.23 ± 0.09	1.20 ± 0.21	4.58 ± 0.47	1.60 ± 0.47	9.60 ± 3.62

All animals were injected with 180–230 kBq/10 pmol peptide; ^1^ Block mice group with animals co-injected with 100 μg NT for in vivo NTS1R-blockade along with PA+Lis; ^2^ PA mice group with animals co-injected with 300 μg PA to in situ inhibit NEP; ^3^ Lis mice group with animals co-injected with 200 μg Lis to in situ inhibit ACE; ^4^ PA+Lis mice group with animals co-injected with 300 μg PA and 200 μg Lis to in situ inhibit both NEP and ACE.

**Table 3:** Biodistribution data for ^99m^Tc-DT5, expressed as %ID/g mean ± sd, *n* = 4, in WiDr xenograft-bearing SCID mice at 4 h pi without or with coinjection of PA+Lis.

Tissue	^99m^Tc-DT5 – 4 h pi		
	Block ^1,2^	Controls	PA+Lis ^3^
Blood		0.45 ± 0.16	0.41 ± 0.12
Liver		0.90 ± 0.20	1.12 ± 0.27
Heart		0.16 ± 0.04	0.22 ± 0.05
Kidneys		8.63 ± 1.80	16.62 ± 1.63
Stomach		2.00 ± 0.35	2.02 ± 0.37
Intestines	0.42 ± 0.01	2.90 ± 0.28	4.30 ± 0.52
Spleen		0.31 ± 0.09	0.86 ± 0.07
Muscle		0.07 ± 0.02	0.11 ± 0.02
Lungs		0.43 ± 0.10	1.10 ± 0.16
Pancreas		0.16 ± 0.04	0.29 ± 0.03
Tumor	0.13 ± 0.01	0.88 ± 0.08	12.29 ± 2.73

All animals were injected with 180–230 kBq/10 pmol peptide; ^1^ Block mice group with animals co- injected with 100 μg NT for in vivo NTS1R-blockade; ^2^ values adopted from [23]; ^3^ PA+Lis mice group with animals co-injected with 300 μg PA and 200 μg Lis to in situ inhibit both NEP and ACE.

**Table 4:** Biodistribution data for ^99m^Tc-DT6, expressed as %ID/g mean ± sd, *n* = 4, in WiDr xenograft-bearing SCID mice at 4 h pi without or with coinjection of PA, Lis, or PA+Lis.

Tissue	^99m^Tc-DT6 – 4 h pi				
	Block ^1^	Controls	4 h PA ^2^	Lis ^3^	PA+Lis ^4^
Blood	0.11 ± 0.09	0.10 ± 0.06	0.10 ± 0.03	0.08 ± 0.03	0.09 ± 0.00
Liver	0.36 ± 0.06	0.34 ± 0.07	0.42 ± 0.05	0.28 ± 0.01	0.45 ± 0.07
Heart	0.09 ± 0.04	0.08 ± 0.02	0.12 ± 0.03	0.07 ± 0.02	0.10 ± 0.02
Kidneys	10.83 ± 3.26	6.09 ± 1.15	11.14 ± 3.87	6.24 ± 1.56	14.61 ± 3.16
Stomach	0.93 ± 0.30	0.44 ± 0.11	0.87 ± 0.22	0.62 ± 0.22	0.89 ± 0.16
Intestines	0.46 ± 0.21	1.61 ± 0.55	2.52 ± 0.40	1.46 ± 0.32	2.81 ± 0.32
Spleen	0.48 ± 0.16	0.64 ± 0.14	1.71 ± 0.26	0.75 ± 0.09	1.84 ± 0.28
Muscle	0.03 ± 0.01	0.04 ± 0.03	0.05 ± 0.02	0.03 ± 0.01	0.09 ± 0.06
Lungs	0.54 ± 0.07	0.22 ± 0.06	1.26 ± 0.15	0.19 ± 0.03	1.27 ± 0.02
Pancreas	0.07 ± 0.04	0.10 ± 0.04	0.14 ± 0.02	0.07 ± 0.01	0.16 ± 0.02
Tumor	0.38 ± 0.15	1.68 ± 0.28	3.89 ± 0.71	2.15 ± 0.43	3.50 ± 0.34

All animals were injected with 180–230 kBq/10 pmol peptide; ^1^ Block mice group with animals co-injected with 100 μg NT for in vivo NTS1R-blockade along with PA+Lis; ^2^ PA mice group with animals co-injected with 300 μg PA to in situ inhibit NEP; ^3^ Lis mice group with animals co-injected with 200 μg Lis to in situ inhibit ACE; ^4^ PA+Lis mice group with animals co-injected with 300 μg PA and 200 μg Lis to in situ inhibit both NEP and ACE.
